# Thermal Textile Pixels: The Characterisation of Temporal and Spatial Thermal Development

**DOI:** 10.3390/ma12223747

**Published:** 2019-11-14

**Authors:** Adriana Stöhr, Eva Lindell, Li Guo, Nils-Krister Persson

**Affiliations:** Swedish School of Textiles, Polymeric E-Textiles Research Group, University of Borås, 501 90 Borås, Swedeneva.lindell@hb.se (E.L.); li.guo@hb.se (L.G.)

**Keywords:** smart textile, thermal textile pixel, thermal communication, non-auditory and nonvisual communication, thermal conductivity, Peltier element

## Abstract

This study introduces the concept of a thermal textile pixel, a spatially and temporally defined textile structure that shows spatial and temporal thermal contrast and can be used in the context of thermal communication. A study was performed investigating (a) in-plane and (b) out-of-plane thermal signal behaviour for knitted structures made of three different fibre types; namely, polyamide, wool, and metal containing Shieldex yarn, and two different knitting structures: plain knit and terry knit. The model thermal source was a Peltier element. For (a), a thermography set-up was used to monitor the spatial development of thermal contrast, and for (b), an arrangement with thermocouple measuring temperature development over time. Results show that the use of conductive materials such as Shieldex is unnecessary for the plain knit if only heating is required, whereas such use significantly improves performance for the terry knit structures. The findings demonstrate that the textile pixel is able to spatially and temporally focus thermal signals, thereby making it viable for use as an interface for thermal communication devices. Having well-defined thermal textile pixels opens up potential for the development of matrices for more complex information conveyance.

## 1. Introduction

Thermal communication is perceived through the skin. Textiles are the materials that we often have closest to our skin, which enhances their potential as a medium for thermal communication. Textile materials comprise a vast range of fibres and constructions that possess a wide variety of thermal properties. In order to maximise the functions of smart textiles, it is important to better understand how varying fibre choices and textile constructions affect thermal communication.

**Figure 1:**
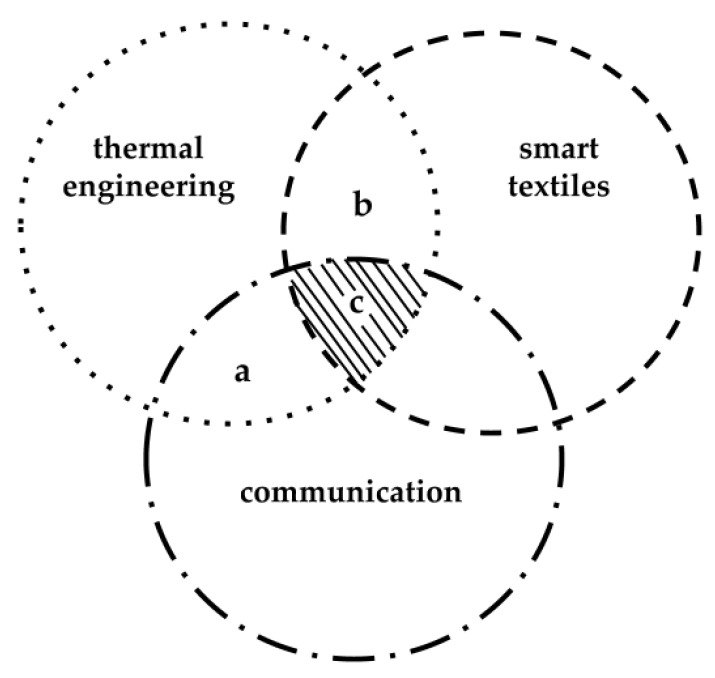
Thermal pixels are at least an intersection between three different technological areas.

This study introduces the concept of a thermal textile pixel and investigates how fibre properties and textile constructions influence signals sent by a thermal source. The thermal pixel represents a counterpart to pixels for visual communication that uses thermal modes (potentially both heating and cooling) for communication. As illustrated in the area denoted (a) in Figure 1, being the overlapping field of thermal engineering and communication.

Numerous studies have examined the thermal properties of textiles in the context of clothing comfort [1,2,3] and textiles used as thermal insulators [4]. Applications of thermal engineering to smart textiles include devices such as underwear [5], socks and gloves [6] with heating functions. Another example, is the approach used to produce the Embr Wrist band [7], a wearable that consists of a thermal cooling module that is able to cool down or heat up for thermal stimulation. Section (b) in Figure 1 encompasses such devices. Beyond these, a broad range of applications opens up in the area of thermal communication in the intersecting area (c) in Figure 1. One example is Lovelet [8], a wearable device that conveys affection between partners through thermal communication. Research is being made on garments designed to be used as feedback, alerting, or communication systems for daily life use by individuals with deafblindness [9]. Wilson et al. [10] observed that although the research field of thermal feedback for communication remains to be fully explored, the implementation of salient feedback based on thermal input can have immense advantages in noisy and turbulent surroundings compared with vibrating inputs [10]. Lee and Lim investigated heat as a communicatorin the field of haptic technology and robotics [11,12]. They concluded that although there is great potential for using thermal devices to facilitate interpersonal communication, the focus should not be to replace current communication types, but rather to enhance or enrich current means of communication [12].

### 1.1. Thermal Communication

The skin is our most expansive organ; it has a complex structure and is involved in a vast number of bodily functions. Apart from the obvious task of defining a bodily inside and a bodily outside and handling moderate mechanical impact, the skin is also involved in maintaining body temperature, mediating sensation, and protecting us from UV-radiation, among other functions [13].

Sensations are mediated through cutaneous sensory systems that receive tactile, thermal painful, and pruritic information and forward them to the central nervous system [14]. Human skin also has two kinds of thermoreceptors, one of which reacts to heat and the other to cold. Thermoreceptor density varies in different parts of the body, and there are many more thermoreceptors that respond to cold than those responding to heat [14]. Temperature exposure can cause acute pain when the skin reaches temperatures above 45 °C or below 5 °C. The temperature at which pain is initiated varies; some examples indicate that being exposed to temperatures below 17 °C can trigger pain sensations [15]. Skin temperature is distinct from body temperature; it normally remains within 32–35 °C but can vary from 20–40 °C [16].

The threshold for thermal perception (the least detectable noticeable difference) is lower for cooler temperatures than for warmer temperatures, and the thresholds are larger for lower body parts than for the upper body [17].

### 1.2. Thermal Expression

Lee and Lim performed a study on heat as a medium for expression in interpersonal communication [11]. In order to better describe heat in the context of communication, they introduced the term thermal expression, which denotes ‘the activity of controlling a particular amount of heat with intention’ [11]. Based on this definition, they described four expressive elements as representations of thermal expression, namely temperature, duration, temperature change rate, and location [11].

The thermal modality of human sensation is susceptible to illusions, such as the phenomenon known as the *thermal grill illusion* [17], which refers to a sensation of heating or cooling that occurs in absence of any change in actual skin temperature. Research using this phenomenon include experiments conducted by Manasrah et al. [18], Hojatmadani and Reed [19], and Oron-Gilad et al. [20]. The latter used the thermal grill illusion in the efforts to develop the foundation for a tactile language [20].

It seems rational to use thermal illusions for communicating signals, which greatly reduce the inconvenience experienced by the subject, as the receiver’s skin temperature does not have to actually reach the given temperatures every time that a signal is sent.

### 1.3. Defining Thermal Textile Pixel

In order to create a thermal illusion, adjacent devices need to be kept separated such that they nonetheless maintain different temperatures. A thermal contrast is needed whereby one can perceive changes in temperature over a sufficient small distance, over a sufficiently short time period, or both (difference in perception due to varying thermal conductivities of materials is not considered). Thus, the thermal textile pixel is a spatially and temporally defined textile structure that shows spatial and temporal thermal contrast and can be used in thermal communication. It is a device for thermal expression within the repertoire of non-auditory and nonvisual communication (NANV).

**Figure 2:**
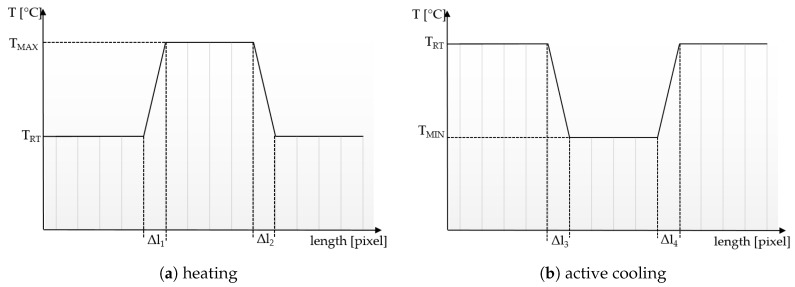
Scheme of a function representing the ideal spatial contrast of a thermal textile pixel. $T_{RT}$ is ambient (room) temperature. (**a**) shows the heating signal, whereby $T_{MAX}$ is temperature sent from thermal source, $T_{MAX}$ should be below 45 °C for avoiding pain. (**b**) shows the active cooling signal, $T_{MIN}$ is temperature sent from thermal source, $T_{MIN}$ should be above 5 °C to avoid pain. Δl:s should be minimally short.

Figure 2a,b illustrate the ideal spatial thermal contrast for both heating and active cooling. The edges should be maximally sharp in order to enable pixel distinction and the temperature T${}_{max}$ resp. T${}_{min}$ should be detectable. Further on, the temperature of the plateaus (T${}_{max}$ resp. T${}_{min}$) should be coherent with the temperature generated by the thermal source.

**Figure 3:**
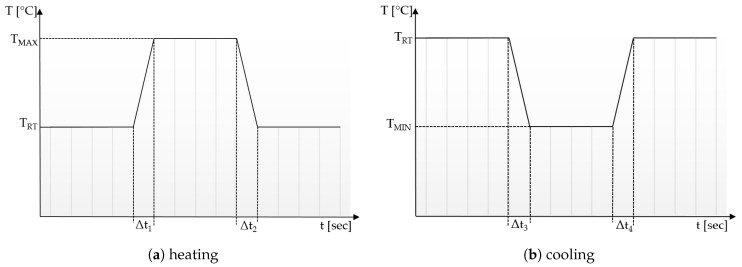
Scheme of a function representing the ideal temporal contrast of a thermal textile pixel. $T_{RT}$ is ambient (room) temperature. (**a**) shows the heating signal, whereby $T_{MAX}$ is temperature sent from thermal source, $T_{MAX}$ should be below 45 °C to avoid pain. (**b**) shows the active cooling signal, $T_{MIN}$ is temperature sent from thermal source, $T_{MIN}$ should be above 5 °C to avoid pain. Depending on what is being communicated Δt:s could be either short or long. In any case it is important that they are controllable.

Figure 3a,b show the ideal temporal thermal contrasts for heating and active cooling, respectively. The temperatures T${}_{max}$ resp. T${}_{min}$ should be predicted based on the repeatability of temperatures generated by the thermal source. Compared with spatial thermal development, in which Δl should be very small, for temporal thermal development, we need to control Δt, meaning adherence to thermal source.

**Figure 4:**
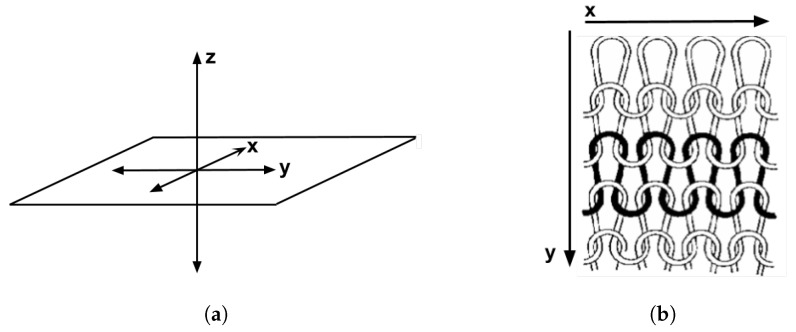
Overview of the in-plane and out-of-plane directions. (**a**) z-direction out of plane, x- and y-direction in plane. (**b**) y denotes the wale direction, x represents the course direction. Figure from Peterson [21].

This study aimed to investigate the effect of textiles on (1) the thermal expression by spatial thermal spread in x-, y- and z-direction, and (2) temporal thermal spread in- and out-of-plane (Figure 4). This was done by measuring the heat spread in- and out-of-plane with an IR camera and measuring the temperature change rate of the textile interface with a thermocouple.

Results show that textile interfaces can be used to convey thermal communication signals and there is an anisotropic effect in the temperature spread in the x-y- and z-direction.

## 2. Results

This section presents the thermal conductivity and temporal and spatial thermal development of six different textile samples. Three samples comprised a plain knit structure consisting of 100% wool (WO), polyamide (PA) and a silver-plated polyamide yarn (Shieldex), the other three samples constituted a terry knit structure consisting of 50/50 WO/PA, 50/50 PA/Shieldex and 100% PA.

Focusing on the material and textile behaviours, a simple realisation of a thermal pixel was used with a Peltier element as the thermal source. A Peltier element was chosen due to its ability to generate both heating and cooling, which is central to mapping how the temperature develops and follows the source.

### 2.1. Thermal Conductivity

The results of measuring the thermal conductivity of the different samples is presented in Table 1. As anticipated, wool showed the greatest thermal resistance, as it was the most insulating fibre used for testing. The plain knit Shieldex exhibited the highest thermal conductivity.

### 2.2. Mapping Temporal Thermal Development, Out-of-Plane

Table 2 and Table 3 present the thermal curves of the interface temperatures of the six different samples. Each curve represents a cycle, which for heating consisted of 300 seconds of heating followed by 300 seconds of thermal dissipation. For active cooling, the cycle consisted of 150 seconds of heating and 150 seconds of thermal dissipation. A prerequisite for the thermal textile pixel is to be able to follow (with possible some lag) the thermal source. Table 2 and Table 3 show the out-of-plane temporal thermal development for the six fabric samples.

Mapping temporal thermal development indicates how quickly the thermal pixel device reacts to the thermal source. In an ideal situation, all thermal energy (heat flux) should be instantly transferred through the textiles, and the textile interface should not reduce the thermal detection percentage. In Table 2 and Table 3, the orange curve should overlap with the grey curve. However, the temperature measured at the textile surface (indicated by orange curves) did not reach the intensity given by the thermal impulse/given temperature from the thermal source (indicated by the grey curves). For heating (Table 2 and Table 3 (a), (c), (e)), PA performs best with plain knit surface ((c)), whereas Shieldex performs best with terry fabric ((a)). Wool is the least effective for heating both knitting structures. For active cooling ((b), (d), (f)), Shieldex performed best ((b)), followed by PA. Again, wool was the least effective material for cooling both plain and terry structures.

Another observation is that the temperature measured at the textile/Peltier element interface in some of the heating processes ((a), (c) in Table 2, (c) in Table 3), unexpectedly increased above the given temperature (compare blue and grey curves), which is untenable from an energy perspective. We interpreted the reason as being that the thermal system did not reach equilibrium because the temperature was measured at 300 seconds after the Peltier element was turned on. The thermal flux was trapped by the textiles and did not complete the process of thermal energy exchange (heat transfer) with the surroundings. The difference between the two curves (blue and grey) is more significant in the case of wool than the other materials, which indicates that the thermal system reaches the equilibrium state much slower in the presence of that material.

### 2.3. Mapping Thermal Spatial Development, In-Plane

Infrared thermography was used to record the temperature every 60 seconds during a 300 second test for heating and at 60 seconds and 150 seconds for active cooling. The results of the spatial development are shown in Table 4 for plain knit and Table 5 for terry knit.

All samples show a bump-like behaviour with relative sharp symmetric ramp up and ramp down. A difference is observable between time to reach T${}_{max}$ (T${}_{min}$) as well as in the evenness of the plateaus.

The Shieldex curves ((a), (b), Table 4 and Table 5) have a wider plateau, and the temperature spreads more in-plane than for the other two non-conductive materials, thus indicating that conductive materials are not favourable for thermal pixels. The same phenomenon can be seen in the case of the terry fabrics (Table 5). Thermal development is less significant for active heating than for heating.

An asymmetry was observed in how the temperature distributed in x- and y- directions. Table 6 shows an example of the typical behaviour. Both x and y showed bump-like behaviour; however, y was typically more blurred.

## 3. Discussion

This work served as an exploratory rather than a comparative study and focused exclusively on investigating heat transfer via textile thermal pixels (therefore excluding psychophysical measurements). Thermal pixels are defined within the context of this work as thermal feedback (both heating and cooling) providing systems consisting of a thermal source paired with a textile component. The thermal output was generated by a Peltier element as a model.

The results provide insight on the impact the textile would have on the elements of thermal expression defined by Lee and Lim [11]: (a) temperature; (b) duration; (c) temperature change rate; and (d) location. Temperatures (a) above detection limits and below pain limits (T${}_{MAX}$ and T${}_{MIN}$) could clearly be reached. Durability has been shown to be achievable for a heating device, whereas an arrangement with a heat sink is necessary for active cooling if a thermoelectrical mechanism is used. In order to convey the ‘correct’ signal, the textile must follow temperature rises and drops with minimal delay (c) of the thermal source or be a consistent function of the thermal source, which also seems feasible. The present study worked with knitted samples rather than readymade garments, and knitting (rather than weaving) was the process of choice. Garments worn close to the body are typically knitted and can be used at almost any body location (d) except the face.

For plain knitting, the use of conductive materials such as Shieldex is unnecessary if only heating is required; however, the use of conductive material significantly improves the performance in the case of terry fabrics. The results presented in Table 2 and Table 3 indicate that signals conveyed with active cooling tend to be less distinct than those conveyed with heating; this is most evident in the case of wool and polyamide. Temporal thermal development results for the terry knit samples (Table 3) confirm that such textile structures with more air entrapment are worse conductors. Textile thermal pixel designs should therefore consider both the thermal properties of the fibre and the textile surface structure.

It is plausible that thermal illusions will serve as the basis for thermal communication, as they enable sending signals without altering the skin temperature of the receiver, which could cause discomfort if occurring over a lengthy duration. Constructing such displays for thermal illusions demands the ability to create adjacent but distinguishable areas that can be kept at different temperatures, such as the thermal grill illusion used by Oron-Gilad et al. [20]. As such, thermal spatial resolution is important. Table 6 shows the presence of a certain blurriness of approximately 20%, which is still below what is needed for the Grill effect. It might be interesting to create displays of several textile pixels for sending more complex messages without any use of thermal illusions, in which case the thermal resolutions of both the material and the human should be considered.

The spatial development shows a discrepancy for heat transfer in the x- and y-directions, with a less conductive tendency in the wale direction of the fabric. This could be explained by the structure of the knitted surface and that there is more air entrapment in the wale directions, thus causing an isolating effect.

Time constants seem long for heating and especially for active cooling. Even the quickest plain Shieldex samples did not reach 90% of the max temperature until 100 seconds, and heat dissipation took 200 seconds (Table 2), which resulted in a total of 300 seconds before the pixel is off again. This indicates a very low updating frequency far below Hz, which is not easily overcome. We conclude that thermal communication should be adapted to the urgency of the message.

## 4. Materials and Methods

This section presents the method, materials, and setups used to measure the thermal conductivity of the textile samples and the measurement of temporal and spatial thermal development.

### 4.1. Textile Materials

Wool is classified as the most thermally insulating fibre [22], whereas polyamides have the best thermal conductivity performance compared to other traditional non-electroconductive fibres. Metals are also known for their outstanding thermal conductivity. Therefore, one conductive yarn was chosen to represent a highly thermal conductivity option. The scattered thermal features of the fibres were expected to yield a wide spectrum of distinguished behaviours.

Two types of textile constructions were similarly chosen for their great differences. The plain knit has a uniform flat face, as opposed to the terry knit, which is more voluminous and porous due to loop formations on the face of the fabric.

Three plain knit samples were made using 100% Shieldex, 100% polyamide and 100% wool. The polyamide was used as a binding thread to achieve a well-functioning fabric in the terry structure, which resulted in a more stable, higher density type of terry fabric. Therefore, the three terry knit samples were made in 50/50 Shieldex/PA, 50/50 WO/PA, and 100% PA. All samples were made with equal stitch lengths of 11.5 on the back side, and the loop was formed with 9.5 stitching on the front and stitch length 8 in the back.

Samples were manufactured on a flat knitting machine: the Stoll type CMS 330 TC multi gauge. Yarn tension was kept stable and a gauge of 12 was maintained throughout the manufacturing procedure. Samples cut with a diameter of 330 mm were used to ensure flat, even fabric during testing.

### 4.2. Characterisation of Thermal Conductivity

Thermal resistance (R) was measured with a two-plate tog-test, in compliance with ISO 5085-1:2004 [23]. The samples’ thickness (d) was measured with a ’Shirley’ thickness gauge by Shirley Development Limited, Manchester according to ISO 5084:1996 [24]. These measurements were necessary in order to 223 calculate the thermal conductivity (k) (Equation (1)) for each sample according to ISO 5085-1:2004 [23].

(1) $$k=\frac{d}{R}\;[W/(m\cdot K)]$$

### 4.3. Characterisation of Heat Source

Prior to the thermal development study, a reference for the ’ideal’ result characterizing the behaviour of the PE only by the mean of thermal outcome from five measurements. All samples were tested at constant room temperature 25 °C. The test samples were exposed to heat and active cooling by an insulated Peltier TEC Module (4), purchased from elektrokit.com [25]. The PE module was provided with power by a dual-tracking DC power supply (9), model TPS-4000 by Topward Electric Instruments Co., Ltd (Hukou, Hsin Chu, Taiwan).

### 4.4. Characterisation of Temporal Thermal Development, Out-of-Plane

**Figure 5:**
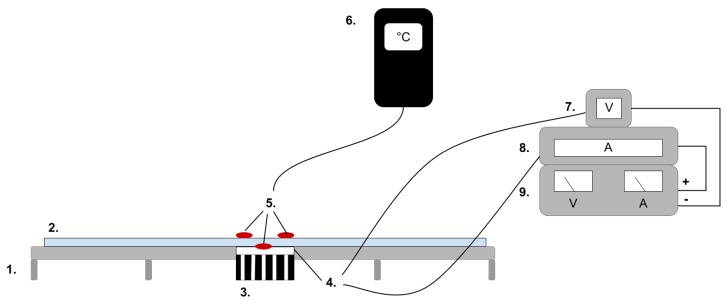
The test setup for the out of plane heat transfer experiment consisted of (1) an elevated board for the sample placement, (2) the textile sample covered by a Styrofoam plate, (3) a heat sink, (4) a Peltier element as thermal source, (5) thermocouples, (6) a data logger thermometer, (7) a voltmeter, (8) a multimeter and (9) a power source.

Prior to the thermal development study, a reference was made characterising the ‘ideal’ behaviour of the PE based on the mean of thermal outcomes from five measurements. All samples were tested at constant room temperature 25 °C. The test samples were exposed to heat and active cooling by an insulated Peltier TEC Module ((4) Figure 5) purchased from elektrokit.com [25]. The PE module was supplied with power by a dual-tracking DC source ((9) Figure 5): model TPS-4000 by Topward Electric Instruments Co., Ltd.

**Figure 6:**
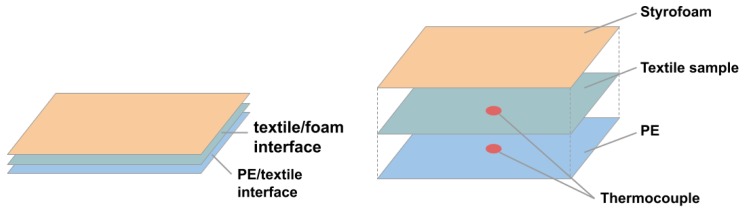
Setup of test specimen for temporal resolution test.

Figure 6 illustrates the instalment of the samples for testing and the placement of the thermocouples. One thermocouple was placed between the Peltier element and textile sample to give insight on the thermal development of the PE/textile interface. The second device was placed on top of the textile sample in the centre of the area directly affected by the Peltier element. Additionally, a Styrofoam plate was placed on top of the setup to keep the thermocouple in place. The third thermocouple recorded the room temperature (RT). The collected data were recorded by a three-channel data logging thermometer (6): model SD 200 by Extech Instruments.

The heating cycle began by exposing the PE to 300 mA of current for 300 seconds before turning off the power supply and letting the system cool down for another 300 seconds. The thermal development after the heat source was turned off is herein referred to as ‘thermal dissipation’. A shorter temporal interval in combination with higher current was necessary for the active cooling cycle, as a preliminary study had showed that the heatsink was not sufficient to thermally balance the high amount of heat generated if a cooling effect was demanded over a longer period of time. Hence, the active cooling generated by changing the polarity of the PE was implemented by applying 500 mA for 150 seconds to cause active cooling, and the thermal development was monitored for another 150 seconds after turning the current off.

The mean data of five test rounds was used throughout the study and plotted to generate time dependent thermal curves of both the PE and the textiles measured with the thermocouple.

### 4.5. Characterisation of Spatial Thermal Development, In-Plane

**Figure 7:**
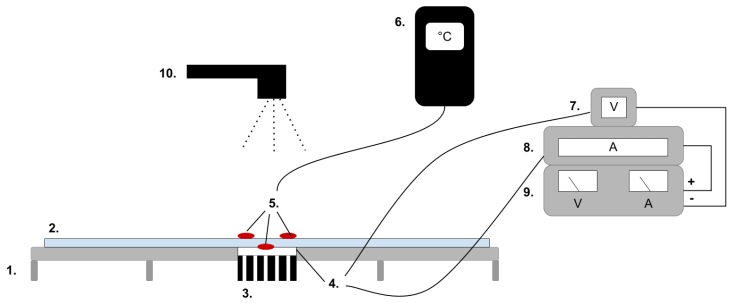
The test setup for the in-plane heat transfer is based on setup in Figure 5 with an the addition of (10) an IR camera (10).

The in-plane heat transfer was investigated via infrared thermatography. The same setup was used as for the temporal resolution except for the addition of an infrared camera E4 by FLIR, which was fixed at a distance of 25 cm above the test setup; see (10) in Figure 7.

The heat spread was recorded every 60 seconds over a period of 300 seconds for heating. For the active cooling cycle, the heat spread was recorded at 60 and 150 seconds.

**Figure 8:**
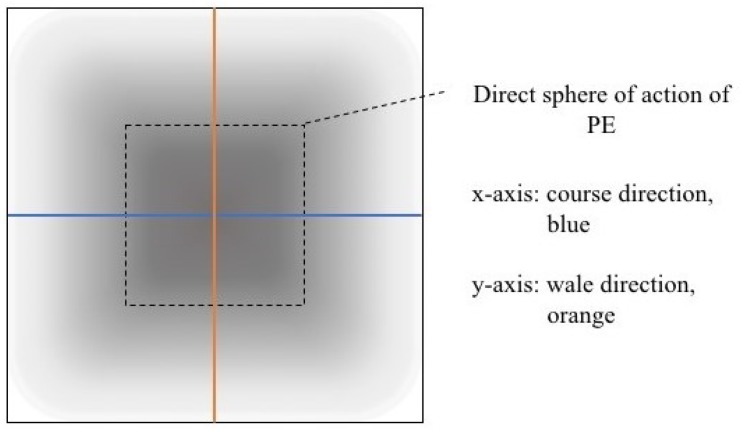
Scheme of IR image, heat spread was made visual by colour gradient and analyszed in the FLIR plus tool.

By using the FLIR plus tool [26] provided by the IR camera manufacturer, the images taken by the IR camera could be analysed using thermal measurements for every pixel of the picture created by the camera. For clarity and to avoid confusion with the thermal textile pixel, the IR camera pixels will hereafter be referred to as area units. Each dataset from the IR camera consisted of 140 area units and their corresponding temperature measurements. By defining two data lines, x- and y-direction corresponding to the course and whale direction of the fabric, it was possible to compare the thermal spread in each direction (Figure 8).

## Figures and Tables

**Table 1:** Measured parameters and thermal conductivity calculated with Equation (1) for each of the six textile samples.

Structure	Material	Mass per Unit Area [g/m${}^{2}$]	Thickness (20 Pa) [mm]	StDv	Air Flow Resistivity (Face) Pa·s/m${}^{2}$	StDv	Thermal Resistance [m${}^{2}$K/W]	Thermal Conductivity [W/(m·K)]
Plain	Shieldex	239.83	1.56	±0.07	49.82	±1.62	0.015	0.10
	PA	261.96	2.01	±0.18	171.4	±1.14	0.024	0.08
	WO	524.05	3.41	±0.13	79.24	±2.67	0.104	0.03
Terry	Shieldex/PA	273.58	3.03	±0.13	78.8	±1.22	0.044	0.07
	PA	354.22	3.02	±0.08	165.4	±0.89	0.035	0.09
	WO/PA	441.52	4.47	±0.03	71.34	±1.39	0.119	0.04

**Table 2:** Temporal thermal development, out-of-plane, for plain knit samples. Measured with thermocouples according to setup in Figure 5. Room temperature (RT) is denoted in black, the orange curve represents the temperature of the textile, and the blue curve represents the temperature of the Peltier element (PE). The light grey curve was measured prior to testing and is equivalent to the thermal energy put into the system, hence representing the ideal outcome for the textile. Temporal thermal development for heating given in (a) plain knit Shieldex sample, (c) plain knit wool sample, (e) plain knit polyamide sample. Temporal thermal development for active cooling given for (b) plain knit Shieldex sample (d) plain knit wool sample, (f) plain knit polyamide sample.

Heating	Active Cooling
plain knit	
Shieldex	
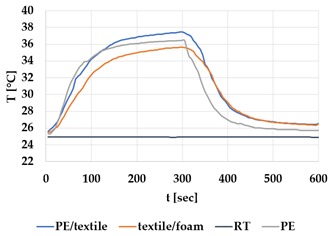 (**a**)	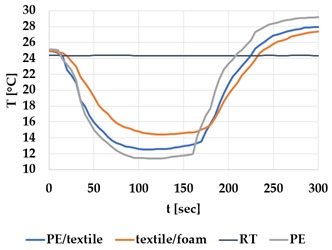 (**b**)
wool	
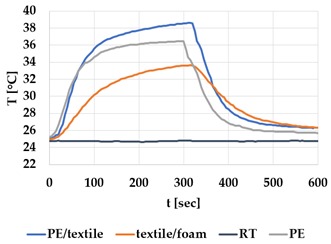 (**c**)	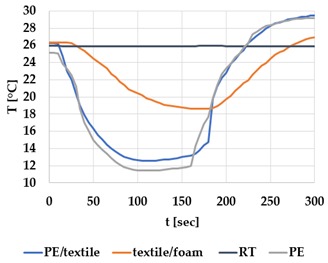 (**d**)
polyamide	
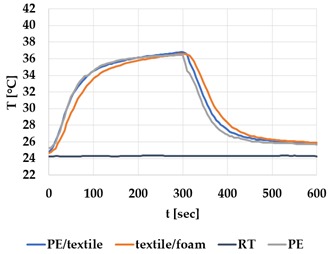 (**e**)	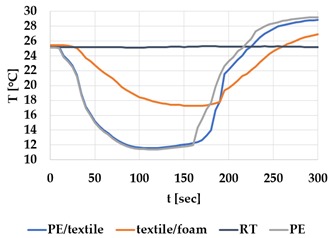 (**f**)

**Table 3:** Temporal thermal development, out-of-plane, for terry knit samples. Measured with thermocouples according to setup in Figure 5. Room temperature (RT) is denoted in black, the orange curve represents the temperature of the textile, and the blue curve represents the temperature of the Peltier element (PE). The light grey curve was measured prior to the testing and is equivalent to the thermal energy put into the system, hence representing the ideal outcome for the textile. Temporal thermal development for heating given in (a) terry knit Shieldex sample, (c) terry knit wool sample, (e) terry knit polyamide sample. Temporal thermal development for active cooling given for (b) terry knit Shieldex sample (d) terry knit wool sample, (f) terry knit polyamide sample.

Heating	Active Cooling
terry knit	
Shieldex/PA	
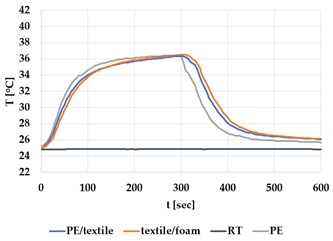 (**a**)	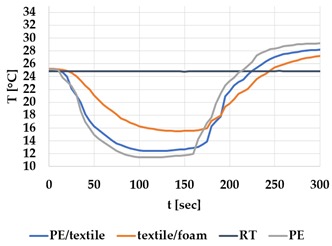 (**b**)
wool/PA	
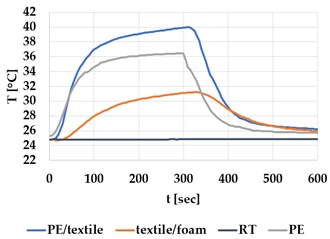 (**c**)	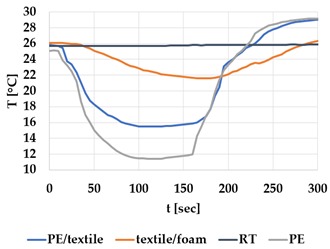 (**d**)
polyamide	
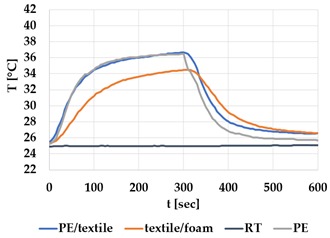 (**e**)	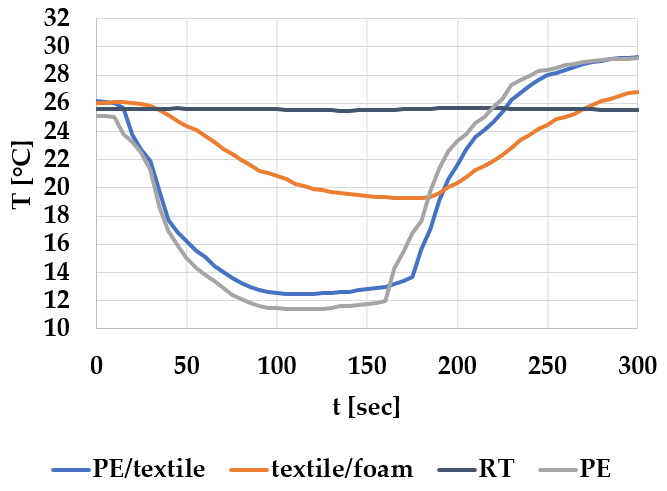 (**f**)

**Table 4:** Spatial thermal development for plain knit samples, results from thermography. (**a**,**b**) plain knit Shieldex, (**c**,**d**) plain knit polyamide, (**e**,**f**) plain knit wool.

Heating		
plain Shieldex	plain polyamide	plain wool
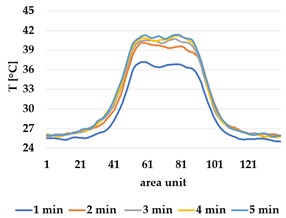 (**a**)	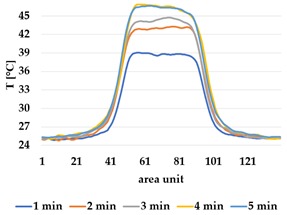 (**c**)	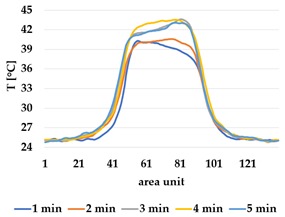 (**e**)
**Active Cooling**		
plain Shieldex	plain polyamide	plain wool
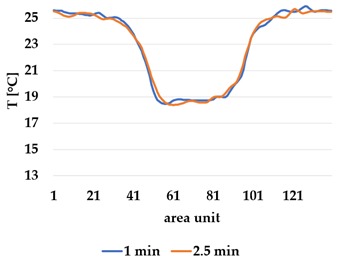 (**b**)	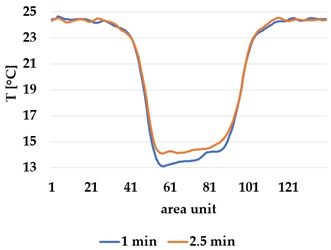 (**d**)	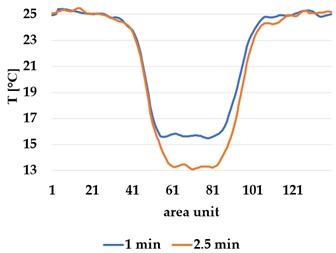 (**f**)

**Table 5:** Spatial thermal development in-plane for terry knit samples, results from thermography. (**a**,**b**) terry knit shieldex, (**c**,**d**) terry knit polyamide, (**e**,**f**) terry knit wool.

Heating		
terry Shieldex/polyamide	terry polyamide	terry wool/polyamide
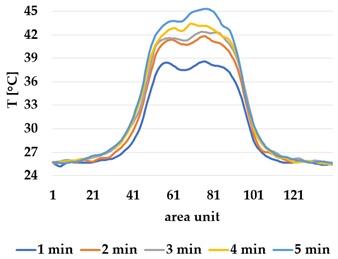 (**a**)	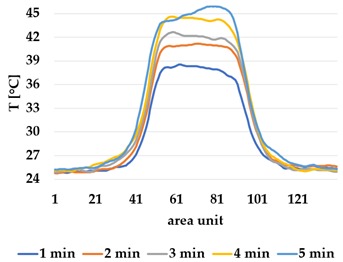 (**c**)	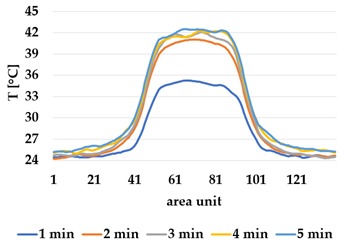 (**e**)
**Active Cooling**		
terry Shieldex/polyamide	terry polyamide	terry wool/polyamide
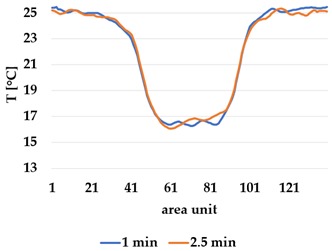 (**b**)	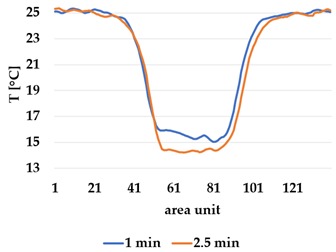 (**d**)	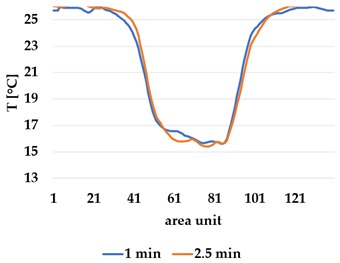 (**f**)

**Table 6:** Directional dependency of spatial thermal development for Shieldex terry knit. (a) directional dependency for heating terry knit Shieldex/polyamide sample. (b) directional dependency for active cooling terry knit Shieldex/polyamide sample.

Heating	Active Cooling
Terry Shieldex/polyamide	
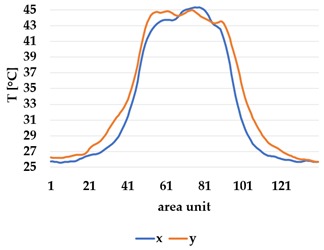 (**a**)	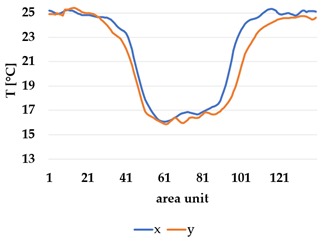 (**b**)
